# The Nuclear Progesterone Receptor Reduces Post-Sigh Apneas during Sleep and Increases the Ventilatory Response to Hypercapnia in Adult Female Mice

**DOI:** 10.1371/journal.pone.0100421

**Published:** 2014-06-19

**Authors:** François Marcouiller, Ryma Boukari, Sofien Laouafa, Raphaël Lavoie, Vincent Joseph

**Affiliations:** Department of Pediatrics and Research Centre CHU de Québec, Université Laval, Québec (QC), Canada; University of Alberta, Canada

## Abstract

We tested the hypothesis that the nuclear progesterone receptor (nPR) is involved in respiratory control and mediates the respiratory stimulant effect of progesterone. Adult female mice carrying a mutation in the nPR gene (PRKO mice) and wild-type controls (WT) were implanted with an osmotic pump delivering vehicle or progesterone (4 mg/kg/day). The mice were instrumented with EEG and neck EMG electrodes connected to a telemetry transmitter. The animals were placed in a whole body plethysmograph 7 days after surgery to record ventilation, metabolic rate, EEG and neck EMGs for 4 consecutive hours. The animals were exposed to hypercapnia (5% CO_2_), hypoxia (12% O_2_) and hypoxic-hypercapnia (5% CO_2_+12% O_2_–5 min each) to assess chemoreflex responses. EEG and EMG signals were used to characterize vigilance states (e.g., wake, non-REM, and REM sleep). PRKO mice exhibited similar levels of minute ventilation during non-REM and REM sleep, and higher frequencies of sighs and post-sigh apneas during non-REM sleep compared to WT. Progesterone treatment increased minute ventilation and metabolic rate in WT and PRKO mice during non-REM sleep. In WT mice, but not in PRKO mice, the ventilation under hypercapnia and hypoxic hypercapnia was enhanced after progesterone treatment. We conclude that the nPR reduces apnea frequency during non-REM sleep and enhances chemoreflex responses to hypercapnia after progesterone treatment. These results also suggest that mechanisms other than nPR activation increase metabolic rate in response to progesterone treatment in adult female mice.

## Introduction

Progesterone has a respiratory stimulant effect [1], [2]. Clinical studies have established that progesterone administration reduces apnea frequency in adult women [3], and progesterone in combination with estradiol treatment after menopause reduces apnea and desaturation events during sleep [4]. Progesterone acts on the hypothalamus [5], nucleus tractus solitarius [6] (NTS - the major site of peripheral chemoreceptor integration in the brainstem), and the peripheral chemoreceptors (mainly localized within the carotid bodies) to increase ventilation under normoxic or hypoxic conditions in cats [2], [7]. The so-called “nuclear” form of progesterone receptors (nPR) is present in these areas [8]–[10]. The nPR is a member of the “steroid receptor super-family”, and it primarily acts as a transcription factor to increase or reduce the expression of target genes [8]. Some experimental results suggest that nPR mediates responses to progesterone injection on phrenic nerve activity in anesthetized cats [5], [6], apnea frequency in adult rats [11], and peripheral chemoreceptor responses to hypoxia in newborn rats [12]. However, we have limited information on the contribution of nPR to the regulation of breathing during sleep, or on the response to the stimulation of the chemoreflex pathway by hypoxia or hypercapnia and there is no characterization of the respiratory phenotype in mice lacking nPR expression. In addition, progesterone also binds to specific membrane receptors [8]. These membrane receptors include 5 distinct proteins from the “membrane progesterone receptor” family, 2 distinct “membrane-associated progesterone receptor components”, and the Na+/K+ ATPase channel [13]–[16]. Structural predictive studies suggest that most of these proteins function as ion channels [16], while membrane progesterone receptors are coupled with intracellular Gi proteins [13]–[16]. Finally, the progesterone metabolite, allopregnanolone, enhances GABA-A receptor activity [17], which inhibits breathing in fetal and newborn rats [18].

The relative contributions of these diverse receptors on respiratory control are unknown. The present study asked whether deletion of nPR in adult female mice alters breathing. To test this hypothesis, we used mice carrying a null mutation of the gene encoding the nPR protein (PRKO mice) and their wild-type (WT) controls [19]. In addition, we postulated that progesterone treatment in PRKO mice would reveal effects on respiratory control if other types of progesterone binding proteins are involved in these responses. WT and PRKO mice were treated for 7 days with progesterone or vehicle to test this hypothesis. We used whole body plethysmography to measure the frequency of sighs (a typical breathing pattern, often followed by apneas) and post-sigh apneas during non-REM sleep (assessed by EEG and EMG) to verify the potential effects of progesterone on breathing stability during sleep. The mice were exposed to hypoxia, hypercapnia, and hypoxic-hypercapnia to estimate chemoreflex responses.

## Materials and Methods

### Animals

This study was performed in strict accordance with the recommendations of the Canadian Council on Animal Care. The committee on the protection of animals of the CHUQ Research Center approved the protocol (Permit Number: 2012-023 for the experimental protocol, and 2012-024 for the colony of PRKO mice). All efforts were made to minimize suffering, reduce the number of animals used, and provide enrichment in housing conditions. WT and PRKO adult female mice (3–5 months old) were obtained from our breeding colony (the founders of the colony were a generous gift from J. Lydon–Baylor College of Medicine – Houston, TX, USA). Homozygous (nPR^−/−^) females exhibit numerous defects in reproductive tissues, including the inability to ovulate, uterine hyperplasia and inflammation, limited mammary gland development, and a lack of sexual behavior [20]. Therefore, the colony was maintained by mating heterozygous females with PR^−/−^ males.

The genotypes of adult female mice were determined before the experiments using genomic DNA extracted by boiling ear punches in an alkaline solution (30 min, 95°C). A total of 1 µl of DNA was used for PCR amplification using a Taq DNA polymerase (New England Biolab, Ipswich, MA, USA). The following primers were used: PrlacZ (5′-ctt cac cca ccg gta cct tac gct tc-3′), P1 (5′-tag aca gtg tct tag act cgt tgt tg-3′), and P3 (5′-gat ggg cac atg gat gaa atc-3′). The presence of the PCR products was detected using agarose (1.7%) gel electrophoresis with a DNA gel stain and DNA ladder. The primer pair of P1 and P3 amplified a 590-bp band in PR^+/+^ mice, and the primer pair of P1 and PRlacZ amplified a 148-bp band in PR^−/−^ mice [19], [21].

### Instrumentation for Sleep Recordings

We used a total of 60 mice for this study, and 30 of them underwent simultaneously recording of breathing parameters and EEG/EMG traces. The other 30 mice were not instrumented for EEG/EMG recordings. One week before recording, the animals were instrumented under isoflurane anesthesia with cranial and neck electrodes connected to a telemetry emitter (PhysioTel F20-EET probe, Data Science International - DSI, New Brighton, MN, USA, bandwidth of transmitted data: 1–50 Hz) placed in the peritoneal cavity for EEG and EMG recordings. All procedures to install the electrodes were performed according to the instructions provided by DSI. Briefly, EEG and EMG electrode wires were routed under the skin from the peritoneal cavity, and the mice were placed on a stereotaxic frame. We inserted screws through the skull+1-mm lateral to midline/+1-mm to bregma, and −1-mm lateral to midline/−3-mm to bregma. The bare ends of EEG electrodes were tightly wrapped around the screws, and the skull and EEG electrodes were covered with dental cement. In this configuration, the caudal electrode over the hippocampus recorded theta oscillation characteristics of REM sleep and exploratory behavior, and the rostral electrode over the frontal cerebral cortex recorded EEG slow waves that are characteristic of NREM sleep [22]. The bare ends of EMG electrodes were placed in the neck muscles at the base of the skull, 2–3 mm apart.

An osmotic pump filled with progesterone or vehicle solution was implanted in a subcutaneous pocket in the back of all the studied animals (Alzet pump model 1007D, reservoir volume 100 µl, flow rate 0.5 µl/hr). The dose of progesterone corresponded to a daily administration of 4 mg/kg for 7 days.

### Recording of Respiratory and Metabolic Parameters

On the day of recording, the animals were housed in a whole-body, flow-through plethysmograph chamber for adult mice (Emka Technologies, Paris, France). The chamber was constantly flushed with room air (250 ml/min), and a sub-sampling pump (75 ml/min) was used to collect out-flowing air. A built-in pneumotachograph measured airflow related to breathing. Before the experiment, the chamber was calibrated via measurement of the airflow generated by the rapid injection of 0.5 ml of air. Water pressure, CO_2_ and O_2_ levels in the out-flowing air were continuously measured using specific gas analyzers (RH-300, CA-10 and FC-10, Sable Systems, Las Vegas, NV, USA) calibrated using a certified dry gas mix (Linde Canada Limité, Vanier, Qc, Canada). The temperature of the plethysmograph chamber was recorded with a thermocouple, and the body temperature of the mouse was recorded via the telemetry probe or rectal temperature measured at the end of the recording. The telemetry signals were transmitted through a receiver and adapter (RPC-1 PhysioTel receiver, DL-10 adapter, DSI) to provide analog signals for EMG, EEG, and animal temperature. All signals were digitized (Micro 1401 data acquisition system, Cambridge Electronic Design, Cambridge, England) and stored on a computer running Spike 2 software (version 7.06 - CED). Measurements of sleep and breathing were performed continuously between 9∶00 and 13∶00. ***Acute respiratory stimuli*** **:** Once the continuous recordings were completed, mice were sequentially exposed to hypercapnia (5% CO_2_), hypoxia (12% O_2_), and hypoxia + hypercapnia for 5 minutes each, separated with normoxia for 5 minutes. Mice were asleep just before exposure, but most mice were awakened by the hypoxia or hypercapnia. We only analyzed portions of respiratory traces during which the breathing trace was regular. The animals were sacrificed, and a sample of blood was collected, kept at room temperature for 5 minutes, and centrifuged for 5 minutes at 13,000 rpm. The serum was immediately separated and stored at −80°C for further analysis. Serum progesterone assays were performed from each sample using in duplicate an enzyme immunoassay kit in accordance with the manufacturer’s instructions (Progesterone EIA kit, ref: 582601, Cayman Chemical, Michigan USA; detection limit 10 pg/ml; 50% sensitivity at 70 pg/ml).

### Data Analysis

#### Determination of sleep/wake states and EEG spectral density analysis

EMG and EEG signals were used to classify sleep/wake states with a sleep scoring script in Spike 2, based on a previous study [23]. The states are scored using epochs of 20 seconds divided into 5-second segments. Each segment is classified as wakefulness, non-REM sleep, or REM sleep, and each epoch is classified according to the predominant score of the segments and neighbor segments, see [23] for details. We reported the % of time spent awake in non-REM sleep, REM sleep, and in non-determined states during the recording. For the analysis of the EEG’s spectral density, the EEG channel was subjected to a Fast Fourier Transform with a Hanning window, and the spectral densities for the following bands were calculated: δ(2–4 Hz); θ(4–7.5 Hz); α(7.5–13.5 Hz); β(13.5–35 Hz) and reported for each state (e.g., Wake, Non-REM, REM sleep).

**Figure 1:**
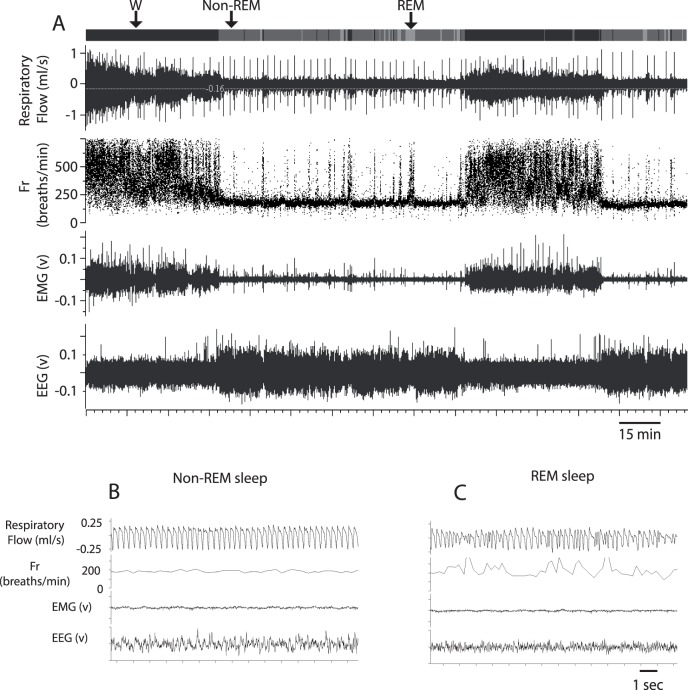
Typical recordings of respiratory flow, instantaneous breathing frequency (breaths/min), EMG, and EEG obtained in 1 animal throughout the recording session (A), during non-REM (B), and REM sleep (C). The upper line in panel A shows the scoring of sleep/wake states (W = wake, non-REM sleep, and REM sleep). Note the numerous sighs that appear as fine peaks during sleep in panel A, and the typical regular breathing pattern in non-REM sleep (B). During REM sleep, the breathing pattern had bouts of irregular activities (C).

Non-REM sleep in mice that were not instrumented with electrodes for EEG/EMG recordings was determined as a long period (>10 minutes) of stable and regular breathing (cf. Fig. 1). REM sleep was determined as brief periods (30–60 seconds) of irregular breathing with higher respiratory frequency and lower tidal volume compared to non-REM sleep. The REM sleep episodes typically occurred during a longer period of non-REM sleep (cf. Fig. 1). There was no difference between the values obtained in each state between the animals in which we determined sleep state using EEG/EMG traces and the animals in which we had no EEG/EMG recordings.

#### Respiratory and metabolic parameters

Episodes of non-REM and REM sleep were selected for each animal. Metabolic parameters and body temperature were not calculated during REM-sleep because most episodes were of short duration, which prevented the adequate equilibration of respiratory gases. Respiratory traces were analyzed to determine the frequency (fR, breaths/min), tidal volume (Vt, ml), and minute ventilation (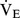 = fR×Vt). Vt was obtained via integration of the negative downward deviations of the flow trace and corrected using a standard equation [24]. Oxygen consumption and CO_2_ production rates were calculated using the following equations [25]:
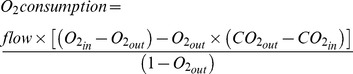


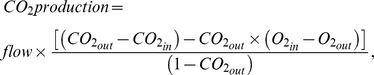
where “flow” is the flow of air measured before entry into the chamber, “O_2in_” and “CO_2in_” are the gas fractions in the inflowing air (considered at 20.9% and 0.038%, respectively), and “O_2out_” and “CO_2out_” are the gas fractions measured in the outflowing line. In these equations, O_2_ and CO_2_ concentrations were corrected with the following term: 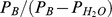, where 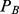 is barometric pressure, and 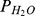 is the partial pressure of water in the inflowing or outflowing air. This correction compensates for the diluting effect of water pressure on measured O_2_ and CO_2_ levels [26]. The ventilatory equivalent for oxygen and carbon dioxide exchange was calculated as (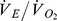
and 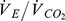). The respiratory quotient (RQ) was calculated as CO_2_ production/O_2_ consumption. Tidal volume, minute ventilation, oxygen consumption and CO_2_ production were expressed as units per 100 g body weight.

Respiratory responses to hypoxia and hypercapnia were analyzed 3–5 minutes after the onset of exposure by selecting periods of stable breathing patterns without movements (assessed by EMG and a stable breathing pattern). All responses were normalized as a % increase compared to a baseline value measured before the exposure to test gas.

#### Sigh and post-sigh apnea frequency

Sighs are characterized by a profound inspiration with a volume that is at least twice the normal tidal volume, and a rapid expiration that can be followed by apneas (2 missed breath) [27]. We determined the frequency of sighs and of post-sigh apneas as performed previously in newborn rats [28]. We selected a period of non-REM sleep lasting 20 to 30 minutes that contained approximately 10 sighs for each animal. Post-sigh apneas are defined as a breath with a frequency at least 50% below the mean value of the corresponding group (i.e., 2 missed breaths) that occur a few seconds after the sigh. We reported the mean duration of apnea (in seconds), the frequency of post-sigh apnea (#/hour of non-REM sleep), and the total time spent in apnea (seconds/hour of non-REM sleep).

### Statistical Analysis

We used GraphPad Prism software (version 6.0c for Mac OSX) for all analyses. All values are reported as the means ± sem, and the significant P value was set a 0.05. P values are reported in the figures with the following general pattern: *, **, ***, and **** are used to report P<0.05, 0.01, 0.001, and 0.0001, respectively.

Two-way ANOVA with repeated measures were performed with group and sleep-state as independent variables. We used one-way ANOVA for values analyzed during one sleep state (metabolic rate). For the EEG power spectrum densities, for each sleep state we performed a two-way ANOVA using frequency band and group. All ANOVAs were followed by Fisher’s LSD test to address specific group effects within each state or the effects of sleep within each group.

## Results

**Figure 2:**
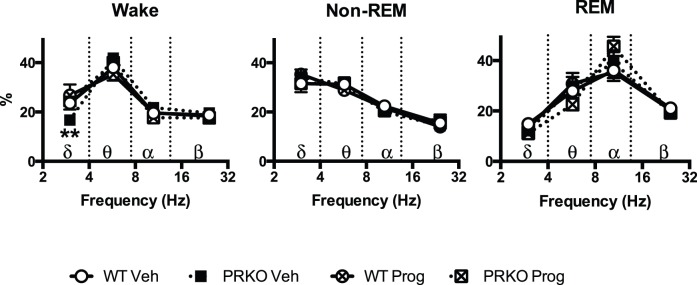
EEG power density (%) during wakefulness, non-REM, and REM sleep in Wild-Type (WT) or PRKO mice treated with vehicle (Veh) or progesterone (Prog) for 7 days. EEG power bands are as follows: δ(2–4 Hz); θ(4–7.5 Hz); α(7.5–13.5 Hz); β(13.5–35 Hz). **: P<0.01 for PRKO mice treated with vehicle vs. other groups.

Table 1 reports the total number of animals used for each group, their body weight, serum progesterone level one week after the implantation of the osmotic pump, and the time spent in each vigilance state. Figure 1 shows a typical recording with the respiratory trace, EEG, EMG and sleep/wake-state channels, and examples of recordings in each vigilance state. There was a significant interaction between time spent in each vigilance state and group (P = 0.03). Compared to WT mice, PRKO mice spent less time awake, and more time in non-REM sleep. Progesterone treatment increased the time spent in non-REM sleep in WT, but not in PRKO mice. During wakefulness, the power density of the low frequency δ band was slightly lower in PRKO mice treated with vehicle compared to other groups (P value for group x frequency band = 0.01 - Figure 2). No other group differences were apparent for the power densities of the EEG signal. There was no difference between groups on the time spent in each state (see Table 1).

**Table 1:** Body weight (BW, g), serum progesterone concentration (ng/ml), % time spent awake, non-REM, and REM sleep during recordings of respiratory variables.

	WT Veh _(n = 12)_	WT Prog _(n = 18)_	PRKO Veh _(n = 17)_	PRKO Prog _(n = 13)_
**BW**	26.1±0.8	26.3±0.6	27.0±1.2	26.3±1.2
**Progesterone**	11.1±2.9	22.7±5.6^0.06^	9.3±3.7	9.5±3.1
**Awake**	61.0±3.3_ (n = 9)_	52.9±3.7 _(n = 5)_	51.2±3.6 _(n = 9)_ #	49.0±5.7 _(n = 7)_
**Non-REM**	28.6±3.8	39.7±3.6*	40.6±2.8#	37.6±6.6
**REM**	2.8±0.9	2.0±0.4	3.3±0.7	3.9±1.1

WT: wild-type mice. PRKO: knock out mice for the nuclear progesterone receptor, Veh: mice treated with vehicle, Prog: mice treated with progesterone. Number of animal in each group indicated as (n = ). Numbers in the “awake” line indicate the number of animals instrumented for EEG/EMG recordings.

*P<0.05 for progesterone vs. vehicle in the corresponding group (WT).

# p<0.05 PRKO vs. WT.

### Respiratory and Metabolic Variables during non-REM and REM Sleep in WT and PRKO Mice following Vehicle or Progesterone Treatment

**Figure 3:**
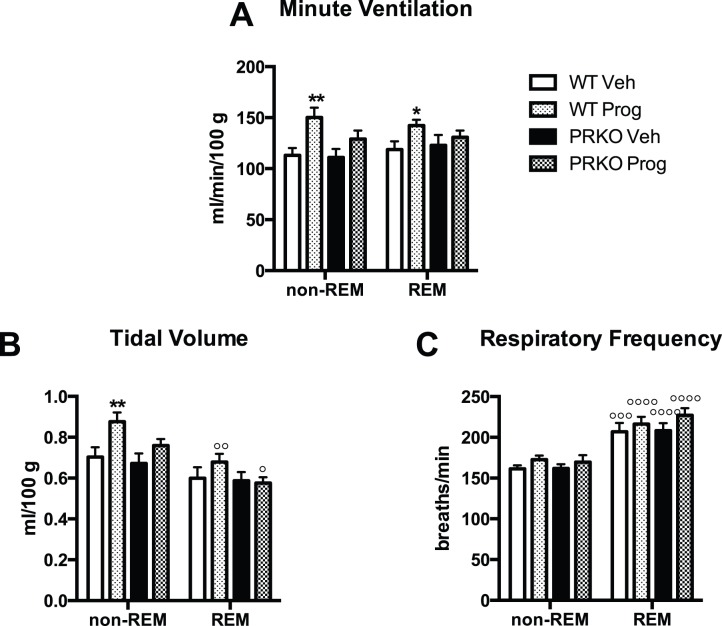
Respiratory parameters recorded during non-REM and REM sleep in WT or PRKO female mice treated with vehicle (Veh) or progesterone (Prog). **A:** Minute ventilation (ml/min/100 g), **B:** Tidal volume (ml/100 g), **C:** Respiratory frequency (breaths/min). *, and **, respectively P<0.05 and 0.01 for the effect of progesterone treatment in WT or PRKO mice. °, °°, °°°, and °°°°: respectively P<0.05, 0.01, 0.001 and 0.0001 REM sleep vs. non-REM sleep.

Progesterone treatment increased minute ventilation in WT mice during non-REM and REM sleep (Figure 3A–P value for group = 0.0007). During non-REM sleep, the value of minute ventilation was 113±7 ml/min/100 g in WT mice treated with vehicle and 150±10 ml/min/100 g in WT mice treated with progesterone (P = 0.002). This effect was due to a higher tidal volume in WT mice treated with progesterone (0.88±0.05 ml/100 g vs. 0.70±0.05 ml/100 g in non-REM sleep – P = 0.006, Figure 3B). No significant effect of progesterone treatment was observed in PRKO mice for minute ventilation despite a tendency towards higher values during non-REM sleep (129±8 ml/min/100 g vs. 111±8 ml/min/100 g, P = 0.07). Minute ventilation was not different between non-REM and REM sleep, but tidal volume was lower and respiratory frequency was higher in REM sleep compared to non-REM sleep (P<0.0001 for both values). The post-hoc analysis showed that tidal volume declined significantly only in WT and PRKO mice treated with progesterone. This result indicates that progesterone specifically increases tidal volume during non-REM sleep compared to REM sleep in WT mice and PRKO mice.

**Figure 4:**
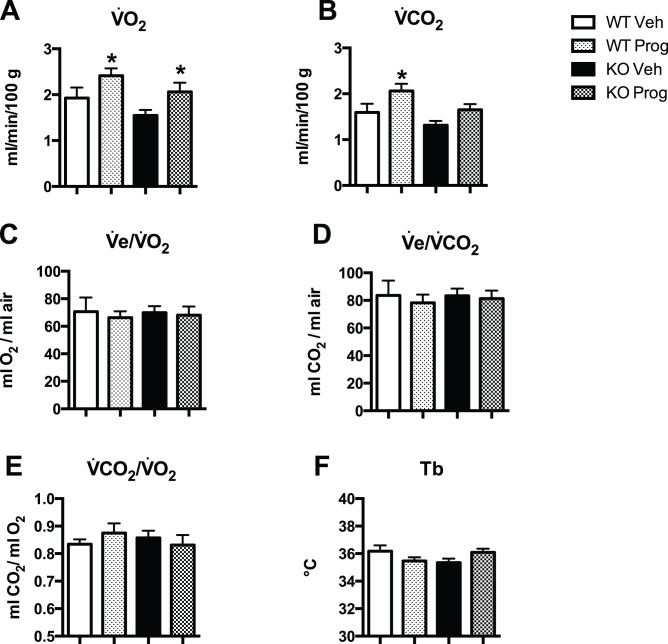
Metabolic parameters recorded during non-REM sleep in WT or PRKO female mice treated with vehicle (Veh) or progesterone (Prog). **A:** Oxygen consumption, **B:** CO_2_ production rate (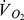 – 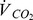: ml/min/100 g - STPD), **C:** oxygen, **D:** CO_2_ convection ratio (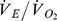
–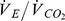), **E:** respiratory quotient (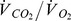), and **F:** body temperature (Tb: °C). *: P<0.05 Progesterone vs. vehicle treatment.

Progesterone treatment increased 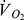 and 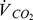 in WT mice (P value for group = 0.004 and 0.002, respectively, Figures 4A–B). 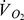 was 1.93±0.23 ml/min/100 g in WT mice treated with vehicle, and 2.41±0.16 ml/min/100 g in WT mice treated with progesterone (P = 0.05). 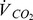 was 1.59±0.19 and 2.06±0.16 ml/min/100 g in WT mice treated with vehicle and progesterone, respectively (P = 0.03). Progesterone treatment increased 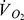 in PRKO mice (P = 0.04) but not 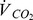 (P = 0.09). There was no group effect for the oxygen or CO_2_ convection ratios, body temperature, or the respiratory quotient (Figures 4 C–F).

### Sigh Frequency during non-REM Sleep in WT and PRKO Mice following Vehicle or Progesterone Treatment

**Figure 5:**
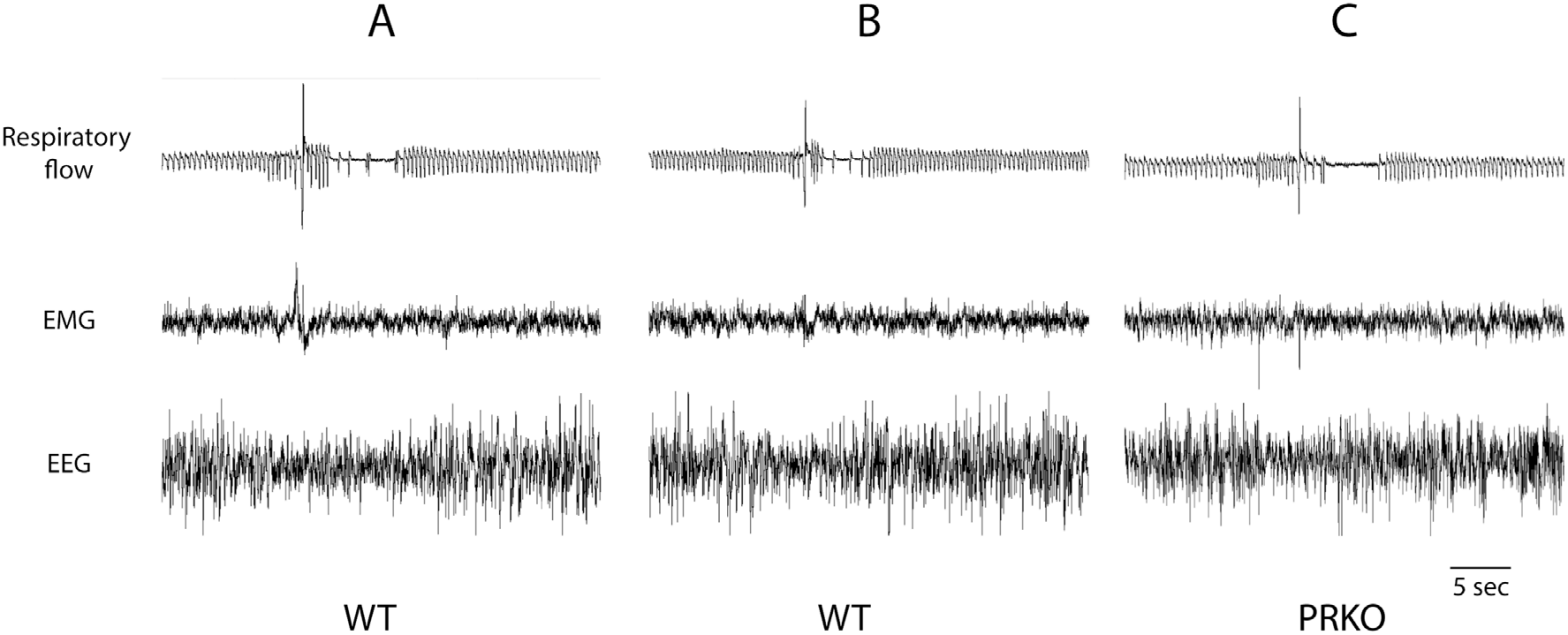
Representative examples of brief wake episodes typically occurring with sigh. **A** (WT mice): Note the irregular breathing pattern, body movements, and reduction of EEG amplitude before the sigh. **B** (WT) and **C** (PRKO): when body movements are not present, the amplitude of the EEG briefly decreases before the sigh. **C:** Note the long post-sigh apnea found in some PRKO animals (see text).

Sighs were characterized by a sequence of events, including a brief pattern of respiratory instability starting a few seconds before the sigh that was generally accompanied by body movements (assessed by EMG) and a brief arousal (assessed by EEG - see Figure 5A). EEG arousal occurred even in the absence of body movements (Figures 5B & C). After the sigh, respiratory frequency declined, and in some cases, apnea or a series of apneas occurred during this period.

**Figure 6:**
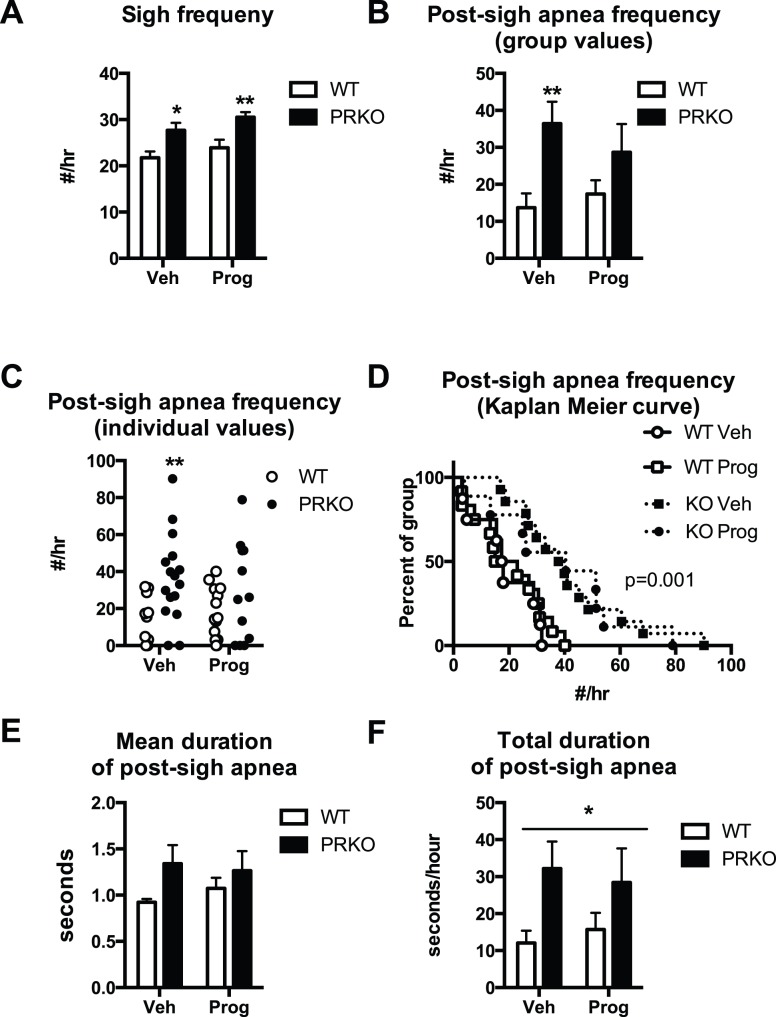
Sighs and post-sigh apneas during non-REM sleep in WT or PRKO female mice treated with vehicle (Veh) or progesterone (Prog). **A:** Frequency of sighs (#/hour of non-REM sleep), **B:** frequency of post-sigh apneas (#/hour–group values), **C:** individual values of frequency of post-sigh apneas, **D:** Kaplan Meier curve of post-sigh apnea frequency. **E:** Mean duration of post-sigh apnea (seconds). **F:** Total duration of post-sigh apnea (seconds/hour). *, and **P<0.05, and 0.01 PRKO vs. WT mice.

Sigh frequency during non-REM sleep was higher in PRKO mice treated with vehicle (27.5±1.7 sighs/hr) compared to WT (21.7±1.4, P = 0.02 - Figure 6A), and in PRKO mice treated with progesterone (30.5±1.1 sighs/hr) than in WT mice treated with progesterone (23.9±1.7 sighs/hr, P = 0.01). The frequency of post-sigh apneas was higher in PRKO mice treated with vehicle (36.4±6 apneas/hr) compared to WT mice (13.7±3.9 apneas/hr – P = 0.006 - Figure 6B). Despite an apparent trend, the difference in apnea frequency between WT and PRKO mice was not significant in progesterone-treated mice (P = 0.16). However, closer examination of the data showed a clear trend of PRKO mice to have a high frequency of post-sigh apnea even after progesterone treatment (Figure 6C). We performed a Kaplan-Meier analysis followed by a Mantel-Cox log-rank test for curve comparisons because the data set of post-sigh apnea values was clearly skewed (Figure 6D). This analysis shows a different pattern of apnea frequency in PRKO mice treated with vehicle or progesterone compared to WT mice.

There was no difference of mean duration of post-sigh apnea (PRKO mice treated with vehicle 1.34±0.2 seconds – range 0.84–3.33) compared to WT mice treated with vehicle (0.92±0.04 seconds - range 0.80–1.09 - Figure 6E-P value for ANOVA = 0.3, post-hoc P value = 0.09). The total duration of post-sigh apnea was not different between groups (P value = 0.11), however if animals are pooled according to genotype, there is a significant difference between WT and PRKO mice (P = 0.02-Figure 6F).

### Hypoxic and Hypercapnic Ventilatory Responses in WT and PRKO Mice following Vehicle or Progesterone Treatment

**Figure 7:**
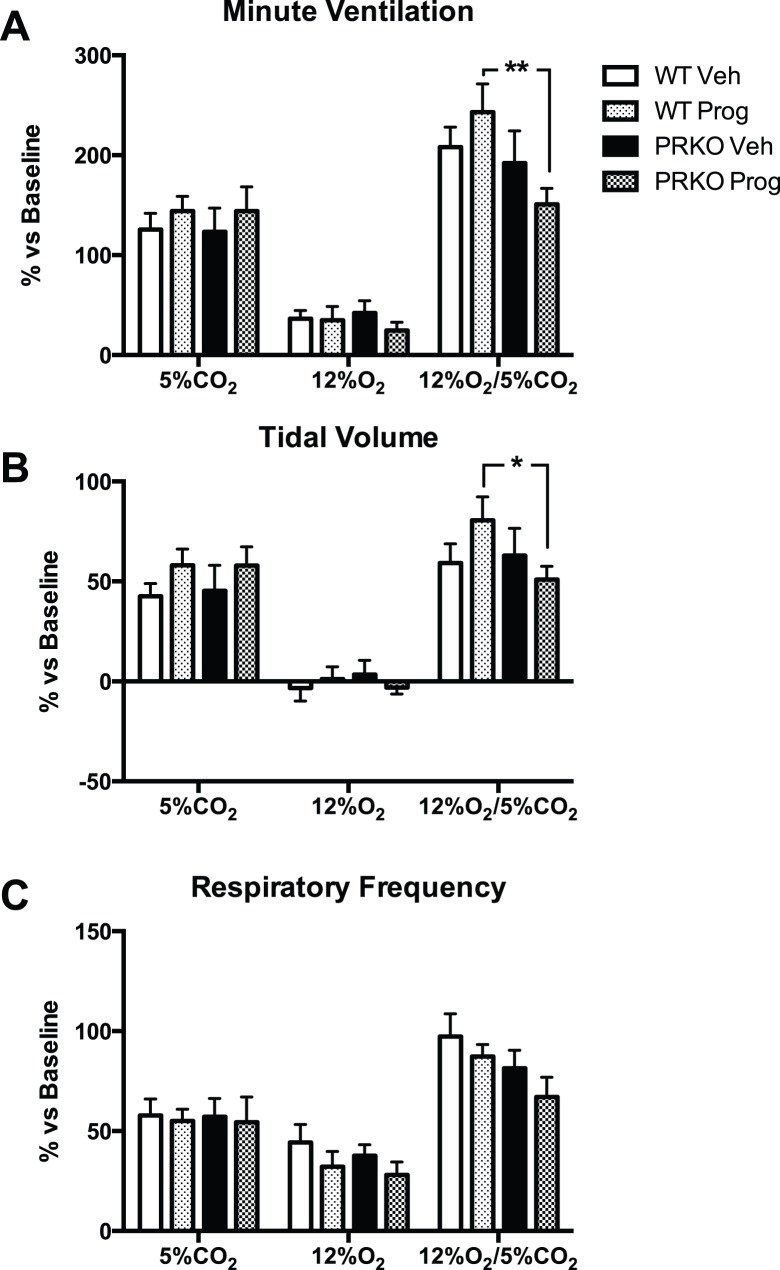
Ventilatory responses to hypoxia (12% O_2_), hypercapnia (5% CO_2_), and hypoxic-hypercapnia (12% O_2_-5% CO_2_) in WT or PRKO mice treated with vehicle or progesterone. **A:** minute ventilation, **B:** Tidal volume, **C:** Respiratory frequency (all % changes vs. baseline). *, **: P<0.05, <0.01, progesterone vs. vehicle.

Absolute values for tidal volume, respiratory frequency, and minute ventilation recorded during exposure to hypercapnia, hypoxia, and hypoxic-hypercapnia are presented in Table 2. Progesterone treatment increased tidal volume (P Value for group effect <0.0001) and minute ventilation (P = 0.0002) under hypercapnia and hypoxic-hypercapnia in WT mice only but had no effect in PRKO mice. Relative (% vs. baseline) changes in minute ventilation, tidal volume, and respiratory frequency in response to each test gas are presented in Figure 7. There was no significant effect of group, but a tendency towards a group x gas level for % Ve and %Vt was observed (P = 0.06 for both values). Post-hoc analyses showed that the ventilatory response to hypoxic-hypercapnia was higher in WT mice treated with progesterone compared to PRKO mice treated with progesterone (Figure 7A for %Ve and 7B for %Vt).

**Table 2:** Tidal volume (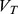: ml/100 g), respiratory frequency (Fr: breaths/min), and minute ventilation (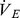: ml/min/100 g) during baseline recordings, and exposure to hypercapnia (5% CO_2_), hypoxia (12% O_2_), and hypercapnic-hypoxia (5% CO_2_+12% O_2_), WT: wild-type mice.

		WT Veh	WT Prog	PRKO Veh	PRKO Prog
		(n = 12)	(n = 17)	(n = 15)	(n = 11)
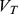	Baseline	0.76±0.07	0.95±0.12	0.71±0.06	0.78±0.04
	5% CO_2_	1.08±0.10	1.44±0.14*	1.01±0.11	1.21±0.05
	12% O_2_	0.73±0.07	0.93±0.10	0.74±0.08	0.76±0.05
	5% CO_2_+12% O_2_	1.21±0.12	1.64±0.17**	1.15±0.12	1.16±0.05
Fr	Baseline	150±4	157±4	159±6	161±7
	5% CO_2_	235±10	243±10	241±12	241±12
	12% O_2_	215±12	207±12	211±10	205±13
	5% CO_2_+12% O_2_	294±15	294±11	286±12	264±10
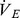	Baseline	114±8	146±15	114±10	124±7
	5% CO_2_	252±24	350±40*	246±36	295±25
	12% O_2_	156±17	201±32	159±19	155±14
	5% CO_2_+12% O_2_	352±41	494±59**	331±46	310±24

PRKO: knockout mice for the nuclear progesterone receptor, Veh: mice treated with vehicle, Prog: mice treated with progesterone. Number of animals in each group indicated as (n = #).

*, and **: P<0.05, and <0.01 respectively for progesterone vs. vehicle in the corresponding group (WT or PRKO).

## Discussion

Our results indicate that deletion of the nuclear PR enhances the frequency of sighs and post-sigh apneas during non-REM sleep and that the nPR is required to enhance ventilatory responses to hypercapnia and hypoxic-hypercapnia after progesterone treatment. Accordingly, this receptor has an important modulatory effect on the regulation of breathing during non-REM sleep, and on respiratory reflexes. On the other hand, progesterone treatment increased metabolic rate and minute ventilation in WT and, to a lesser extent, in PRKO mice during non-REM sleep. These results indicate that the effects of progesterone on the regulation of breathing likely involve different progesterone receptor types, with each receptor type mediating distinct responses.

Serum progesterone concentration was not significantly increased by progesterone treatment. Similar effects have been described previously with chronic steroid treatment in newborn [29] and adult rats [30], which might be a limitation of the ELISA assay when performed on serum samples without prior extraction of steroids. This observation does not imply that biological responses are not elicited by progesterone treatment. The fact that there is no change in progesterone levels in PRKO mice might indicate a higher concentration of serum components that interfere with the assay, but it might also possibly reflect differences of progesterone metabolism or distribution in PRKO mice.

We mainly used EEG and EMG recordings as a tool to properly identify respiratory events during REM/non-REM sleep. However, there are apparent effects of the genotype and progesterone treatment (only in WT mice) on the time spent awake or asleep, and we also noted a small but significant difference on the EEG power spectrum in awake PRKO mice compared to other groups. While these results were obtained on a limited number of animals, this might likely indicate specific effects of the progesterone receptor on sleep homeostasis that could warrant further specific studies.

PRKO mice exhibit signs of uterine inflammation [20], and nPR exerts a potent anti-inflammatory role in the uterus in response to estradiol and progesterone treatment [31]. Accordingly, we cannot completely exclude the hypothesis that some of the effects reported in PRKO mice were due to a local or systemic inflammatory process within the respiratory control system.

The nPR belongs to the superfamily of steroid receptors, and this receptor directly interacts with specific response elements in the promoter regions of target genes [8]. This receptor is found in several parts of the central and peripheral nervous systems that are involved in breathing control, including the carotid bodies [10], nucleus tractus solitarius [9], and the preoptic, paraventricular, ventromedial, dorsomedial, and arcuate nuclei in the hypothalamus [8]. Functional studies support a role for this receptor in respiratory control because the effect of progesterone injection on apneic episodes recorded during sleep (identified by behavioral criteria) in adult rats is abolished by mifepristone (a non-specific antagonist for nPR and glucocorticoid receptors) [11]. Similarly, previous studies in anesthetized cats showed that i.v. progesterone administration enhances phrenic nerve activity. This effect is not elicited by other steroids, and it is blocked by pre-treatment with mifepristone [6]. In this model of vagotomized and chemodenervated cats, the effects of progesterone on respiratory activity is primarily mediated by the hypothalamus [5], but the injection of progesterone in the NTS also increases respiratory activity [6]. Chronic treatment with mifepristone in newborn rats abolished carotid body responses to hypoxia [12], while progesterone treatment in adult cats enhances the carotid body response to hypoxia [7]. Progesterone combined with estradiol in rats enhances ventilation by decreasing the synthesis of the inhibitory neurotransmitter dopamine in the carotid body [32], which is consistent with the effect of the progesterone receptor on the expression of tyrosine hydroxylase [33], the rate-limiting enzyme in dopamine synthesis. While the effects of progesterone on peripheral chemoreceptors would suggest that it increases hypoxic ventilatory response, in adult female rats treated with estradiol + progesterone minute ventilation increases [32], [34], but the effects on hypoxic ventilatory response were small [32]. In adult male rats, progesterone + estradiol treatment increases minute ventilation and hypercapnic ventilatory response [35], and etenogestrel (a potent progesterone receptor agonist) enhances response to metabolic acidosis on in-vitro preparation of the central nervous system in newborn rats [36]. Accordingly, the available findings indicate that in adults progesterone mainly increases ventilation under hypercapnic condition, our data indicate that this effects is mediated by the nuclear progesterone receptor.

An additional feature observed in PRKO mice was the higher frequency of sighs and post-sigh apneas during non-REM sleep. Sighs are part of the normal breathing pattern [37], and they are most likely generated by the activation of pulmonary mechanoreceptors [38] or peripheral chemoreceptors [39], which send afferent inputs into the NTS, which relays these inputs to the central respiratory generator. In the present study, sighs were accompanied by brief awakenings (noted on the EEG and EMG signals), an index of sleep fragmentation and poor sleep quality [40]. Sighs in human infants are followed by skin vasoconstriction, which is an index of sympathetic activation [41]. However, vagal tone increases after the sigh [42]. Spontaneous sighs in adult humans reduce tension in the trapezius muscle and restore autonomic respiratory control, as evidenced by increased structured breathing variability after the sigh (as opposed to higher random variability before) [43]. These findings indicate that sighs engage an array of autonomic processes and help maintain breathing stability. Similarly, our recordings indicate that breathing is irregular before each sigh, but it becomes much more regular after the sigh. It is a common observation in rats [44], mice [27], and human infants [42], [45] that sighs might be followed by an apnea, which is likely an inhibitory response initiated by the activation of pulmonary stretch receptors. Since PRKO mice had higher sighs and post-sigh apnea frequency, the processes regulating this specific respiratory pattern and the responses that are elicited by the sighs are likely regulated by nPR either directly, or indirectly.

Progesterone increased minute ventilation and metabolic rate during sleep in WT and PRKO mice, but 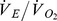 and 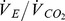 were not affected by progesterone treatment, and therefore, it is likely that the higher ventilation is simply an “adjustment” to the higher metabolic rate. Metabolic rate increases during pregnancy [46], and combined progesterone and estradiol treatment increases metabolic rate in cats [7]. Our results suggest that this effect is not mediated by the nuclear progesterone receptor, but it may involve non-genomic progesterone receptors. Currently, five different membrane progesterone receptors have been identified [13], and these receptors are members of the progestin and adipoQ receptor (PAQR) family. These proteins have 7 transmembrane domains with extracellular and intracellular terminals that confer selective progesterone binding and activation of intracellular Gi proteins [14], [15], [47]. Recent computational structural analyses suggest that these receptors might also function as ion channels [16]. The first reports on these receptors showed a predominant expression of mPRα in reproductive tissues and mPRβ in the brain [48]. In mice, mPRα and mPRβ proteins and mRNAs are expressed in the spinal cord with a distinct staining pattern that likely underlies the important trophic and protective effects of progesterone at this level [21]. The mRNAs for mPRα and mPRβ in rats are expressed in the cortex and thalamic nuclei [49]. In addition, progesterone receptor membrane component 1 (pgrmc1) has been identified in the NTS, and in the pre-Bötzinger complex in adult rats [50]. Finally, a truncated form of the nuclear progesterone receptor that lacks the DNA binding domain is present in mitochondria, and increases oxygen consumption [51]. However if this truncated form of nPR shares the same promoter of the nPR it should also be absent in PRKO mice, so far we have no relevant information on this point. Therefore, these components may also mediate some effects of progesterone on respiratory or metabolic control.

We conclude that the nuclear progesterone receptor plays an important role in respiratory control: it enhances the ventilatory response to hypercapnia and hypoxic-hypercapnia during progesterone treatment, contributes to the reduced frequency of sighs during sleep (and likely reduces sleep fragmentation), and reduces the frequency of post-sigh apneas. However, some effects of progesterone are present despite the absence of this receptor, which implicates different receptors in these effects. The elucidation of the roles of these additional progesterone receptors in respiratory control will require additional experiments, which could open new avenues to explain the respiratory stimulant effects of progesterone.
